# Antibody inhibition of influenza A virus assembly and release

**DOI:** 10.1128/jvi.01398-23

**Published:** 2024-01-05

**Authors:** Yuanyuan He, Zijian Guo, Sofie Subiaur, Ananya Benegal, Michael D. Vahey

**Affiliations:** 1 Department of Biomedical Engineering, Washington University in St. Louis, St. Louis, Missouri, USA; 2 Center for Biomolecular Condensates, Washington University in St. Louis, St. Louis, Missouri, USA; Emory University School of Medicine, Atlanta, Georgia, USA

**Keywords:** influenza virus, antibody, virus assembly, virus egress, cross-linking, computational model

## Abstract

Antibodies are frontline defenders against influenza virus infection, providing protection through multiple complementary mechanisms. Although a subset of monoclonal antibodies (mAbs) has been shown to restrict replication at the level of virus assembly and release, it remains unclear how potent and pervasive this mechanism of protection is, due in part to the challenge of separating this effect from other aspects of antibody function. To address this question, we developed imaging-based assays to determine how effectively a broad range of mAbs against the IAV surface proteins can specifically restrict viral egress. We find that classically neutralizing antibodies against hemagglutinin are broadly multifunctional, inhibiting virus assembly and release at concentrations 1–20-fold higher than the concentrations at which they inhibit viral entry. These antibodies are also capable of altering the morphological features of shed virions, reducing the proportion of filamentous particles. We find that antibodies against neuraminidase and M2 also restrict viral egress and that inhibition by anti-neuraminidase mAbs is only partly attributable to a loss in enzymatic activity. In all cases, antigen crosslinking—either on the surface of the infected cell, between the viral and cell membrane, or both—plays a critical role in inhibition, and we are able to distinguish between these modes experimentally and through a structure-based computational model. Together, these results provide a framework for dissecting antibody multifunctionality that could help guide the development of improved therapeutic antibodies or vaccines and that can be extended to other viral families and antibody isotypes.

**IMPORTANCE:**

Antibodies against influenza A virus provide multifaceted protection against infection. Although sensitive and quantitative assays are widely used to measure inhibition of viral attachment and entry, the ability of diverse antibodies to inhibit viral egress is less clear. We address this challenge by developing an imaging-based approach to measure antibody inhibition of virus release across a panel of monoclonal antibodies targeting the influenza A virus surface proteins. Using this approach, we find that inhibition of viral egress is common and can have similar potency to the ability of an antibody to inhibit viral entry. Insights into this understudied aspect of antibody function may help guide the development of improved countermeasures.

## INTRODUCTION

Influenza A viruses (IAVs) are segmented, negative-sense RNA viruses that assemble at the plasma membrane of infected cells (1). The assembly and budding of IAVs involve the coordinated action of the viral surface proteins hemagglutinin (HA), neuraminidase (NA), and the proton channel M2, along with the internal matrix protein M1. HA, NA, and M2 are each abundantly expressed on the surface of IAV-infected cells, and they are packaged into virions during budding with relative stoichiometry of approximately 100:25:3 (2). Although IAV assembly and release is not fully understood, the viral membrane proteins are thought to play differentiated yet coordinated roles in the process. The receptor binding and fusion protein HA forms clusters in the membrane of infected cells and potentially induces membrane curvature (3, 4). NA cleaves the glycosidic linkage between virus particles and infected cells, allowing the release of virions for subsequent rounds of infection (5). M2 contributes to membrane scission and incorporation of the viral genome into budding particles (4, 6). The essential roles that these proteins play during IAV assembly and budding represent vulnerabilities that could be exploited in the development of antiviral countermeasures, including vaccines and therapeutic antibodies. While a number of antibodies have been identified that can function in this capacity (7–9), it remains unclear how broadly conserved this functionality may be.

Antibodies neutralize influenza viruses through multiple mechanisms, including inhibition of viral attachment, blocking of viral fusion in the late endosome, restricting the assembly process, and activation of cell-mediated effector functions (10). While established assays are available to evaluate some of these functions (e.g., hemagglutination inhibition for antibodies that block attachment and microneutralization assays for antibodies that inhibit entry), other aspects of antibody function, including inhibition of virus assembly and release, are more challenging to measure or predict. As a result, antibody discovery and characterization have traditionally emphasized an important but somewhat narrow subset of protective mechanisms. However, recent work demonstrating the potency of non-neutralizing antibodies in the control of infection highlights the extent to which antibodies can function outside the context of direct neutralization (11), raising the possibility that multi-functionality—the ability to restrict virus replication through multiple, complementary mechanisms—may be common. However, quantitative methods that can independently evaluate the distinct contributions that a broad range of antibodies make toward the restriction of virus replication are needed to determine if this is the case.

To begin addressing these questions, we developed a fluorescence imaging-based method to quantify antibody inhibition of IAV assembly and release that is agnostic to both the antibody and the viral protein it targets. Using this method, we observed that a wide range of antibodies targeting different antigenic sites on HA, NA, and M2 is capable of inhibiting virus release. For antibodies targeting HA, we find that inhibition occurs through the crosslinking of antigens—either on the infected cell membrane or between the viral and cell membrane—in a manner that can be predicted by structure-based models that account for antibody conformational heterogeneity. Inhibition of virus assembly typically occurs at concentrations less than 20-fold higher than the concentrations at which a particular antibody inhibits entry, with some classically neutralizing antibodies that bind to the HA head or the HA stalk inhibiting viral release more effectively than they inhibit viral entry. In addition to reducing the number of viruses released, we find that anti-HA antibodies can also alter the morphology of virions produced during a single replication cycle. Finally, for antibodies that target NA, we find that loss of enzymatic activity accounts for only a portion of their inhibitory effect and that both the potency and mechanism of these antibodies depend on the HA expressed by the target virus. The framework for understanding antibody function described here may be applied to other viruses that assemble at the plasma membrane of infected cells and could help guide the development of vaccines that better elicit multifunctional antibodies.

## RESULTS

### An imaging-based assay to quantify antibody inhibition of viral release

**Fig 1:**
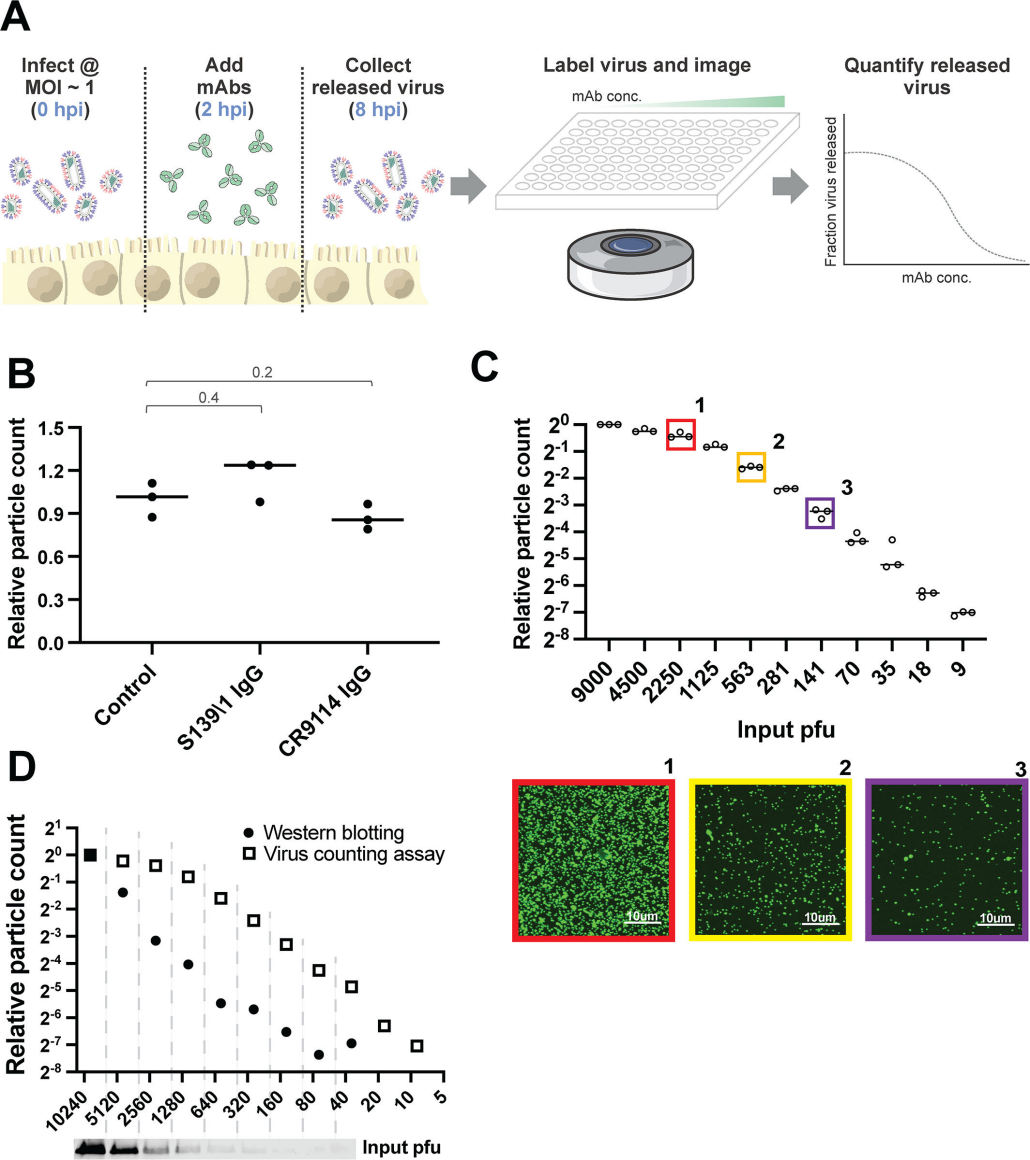
Measuring antibody inhibition of virus release by counting virions. (**A**) Overview of the image-based assay to measure antibody inhibition of virus release. Cells are infected with influenza viruses at MOI ~1 and incubated with monoclonal antibodies (mAbs) begining at 2 hpi. Released virions are collected from the supernatant at 8 hpi, labeled with fluorescent anti-HA single-chain antibody fragments or Fabs that bind to non-competing epitopes relative to the test antibodies, and immobilized for imaging. Segmentation of the resulting images enables quantification of released virions. (**B**) A comparison of virus immobilization on ECL coverslips in the presence or absence of high concentrations of neutralizing antibodies. Antibodies are incubated with virus at a concentration of 60 nM for 30 mins at 4°C before immobilization onto glass-bottom imaging chamber. Data are combined from 3 biological replicates and normalized to the mean of the control conditions. *P* values are determined by Mann-Whitney tests. (**C**) Sensitivity and linearity of virus particle counting compared with quantification from plaque assays. Individual data points are from three separate serial dilutions of A/WSN/1933 virus starting from 3 × 10^5^ pfu/mL. Images below are from the indicated conditions in *C*. Virus particles are visualized using CR9114 scFv labeled with AF488. Contrast in the sample images is exaggerated to show individual virions. (**D**) Comparison of particle counting results to western blot analysis using an anti-HA antibody to quantify released virus. Results from western blot are collected from one serial dilution; results from the imaging-based assay are collected from three sets of serial dilution, and the mean value is shown. Contrast in the western blot scan is exaggerated to show the HA bands.

To determine the potency of antibodies against IAV surface proteins during virus assembly and release, we developed a fluorescence imaging-based approach to directly count virions released into the cell culture media during a single replication cycle (Fig. 1A). Following infection at multiplicity of infection (MOI) ~1, we incubate MDCK cells with monoclonal antibodies starting at 2 hours post-infection (hpi), and we collect viral supernatants at 8 hpi. Released virions are immobilized onto glass-bottom plates coated with *Erythrina cristagalli* lectin (ECL) for fluorescence imaging. This approach is insensitive to high concentrations of HA-specific antibodies (Fig. 1B) and gives linear results across a >100-fold range, from 9 PFU/well (the lowest concentration tested) to 1,125 PFU/well (Fig. 1C). The upper end of this range can be extended arbitrarily by pre-diluting samples for accurate quantification. In addition to authentic virions, the particles detected by anti-HA single-chain variable fragments (scFvs) or Fabs may contain a fraction of HA-containing extracellular vesicles, which we do not account for in the assay. In comparison to western blot analysis, particle counting gave a >10-fold lower limit of quantification (Fig. 1D). Collectively, these results establish fluorescent particle counting as a quantitative and sensitive assay to measure viral shedding in cell culture supernatants in the presence of a range of neutralizing antibodies.

### Monoclonal antibodies targeting a variety of antigenic sites on HA inhibit viral release

**Fig 2:**
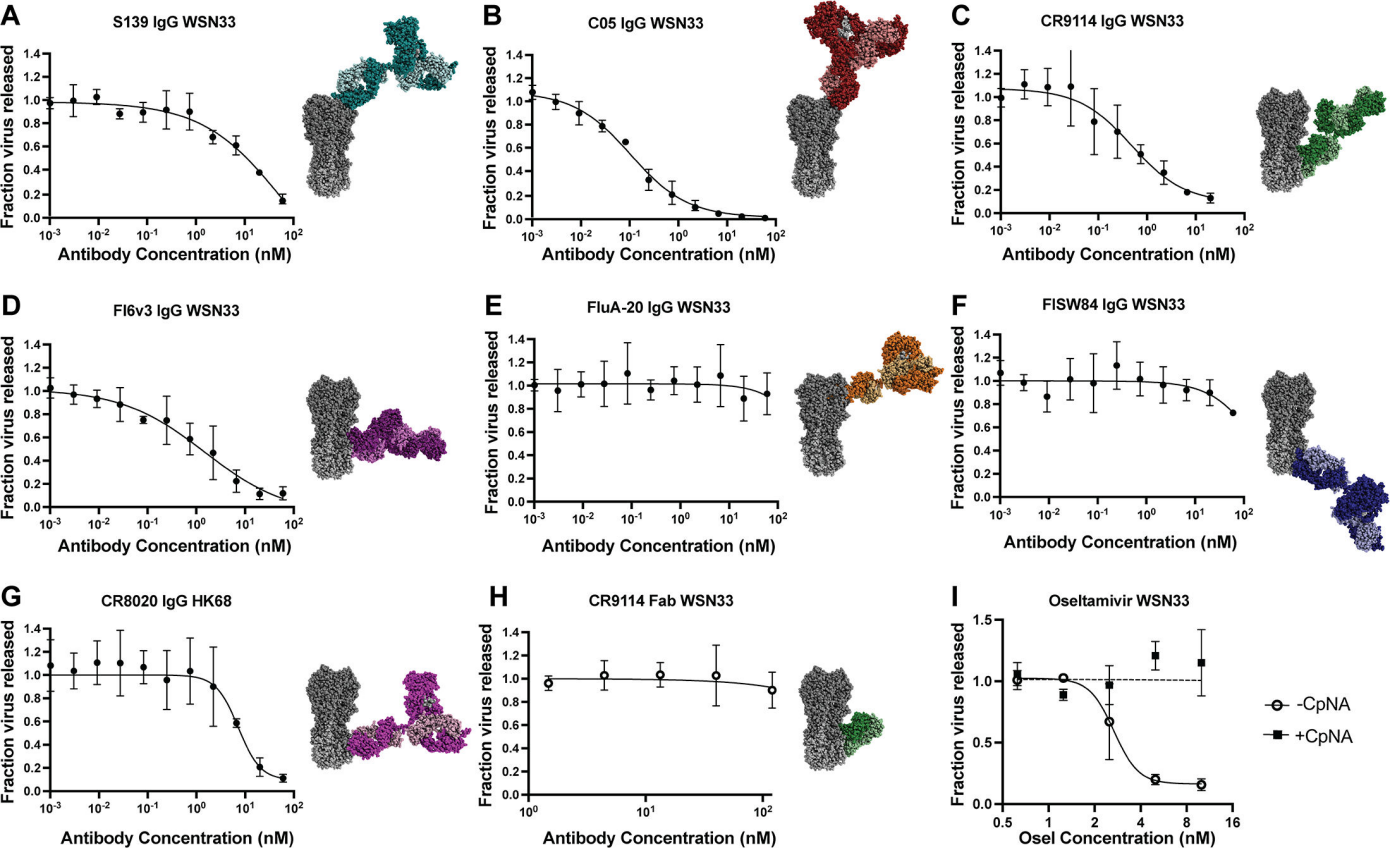
Anti-HA antibodies inhibit influenza virus release from infected cells with supplementation of exogeneous sialidase. (**A–H**) Neutralization curves showing the fraction of viral particles released from cells infected with A/WSN/1933 (“WSN33”) or A/Hong Kong/1968 (“HK68”) as a function of antibody concentration. Each curve is generated from three biological replicates. Error bars show standard deviations, and the fit curves are generated by the least squares method. Images to the right of each plot show HA (Protein Data Bank [PDB] ID 3LZG) in gray with models of full-length IgG1 antibodies bound. Models are obtained by aligning an IgG1 structure (PDB ID 1HZH) to HA:Fab structures (PDB IDs 4GMS, 4FQR, 4FQI, 3ZTJ, 6OC3, 6HJP, and 3SDY). Antibody heavy chains are shown in darker shades with light chains shown in lighter shades. The model of FluA-20 in panel *E* contains clashes with the closed HA trimer. (**I**) Inhibition of viral egress by oseltamivir carboxylate, with or without supplementation of exogeneous sialidase. Data are combined from three biological replicates. Dashed line is added as a guide to the eye. Error bars show standard deviations, and the fit curve is obtained using the least squares method.

Using this method, we tested the effect of anti-HA antibodies on viral release. HA is the most abundant viral membrane protein on both virions and infected cells, and antibodies targeting a range of sites across the HA surface have been identified and characterized. We selected seven anti-HA antibodies targeting the receptor-binding site (RBS) [S139\1 (12, 13) and C05 (14)]; the central stalk [CR9114 (15), FI6v3 (16), and CR8020 (17)]; the trimer interface [FluA-20 (18, 19)]; and the anchor epitope [FISW84 (20, 21)] (Fig. 2). These antibodies are broadly reactive, allowing us to compare results between historic strains from different subtypes: A/WSN/1933 (H1N1) and A/HK/1968 (H3N2). We expressed and purified these antibodies as IgG1 isotypes (differing only in their VH and VL domains) and tested their ability to inhibit virion assembly and release. For these experiments, we tested antibody concentrations up to 60 nM, similar to the serum concentration of a dominant clonotype post-vaccination (22).

Antibodies targeting the HA head (S139\1, C05) or stalk (CR9114, FI6v3, and CR8020) all showed robust inhibition of viral shedding (Fig. 2A through D and G). Inhibitory profiles of these antibodies against filamentous (“WSN 33 M1Ud”) and non-filamentous (“WSN33 WT”) strains that differ only in their M segment are largely similar (Fig. S1A, Methods). In contrast, these antibodies have greater potency against non-filamentous strains during viral entry (Fig. S1B), consistent with previous findings (23). Antibodies that bind to the HA stalk have been shown to inhibit NA activity through steric hindrance (24). In testing each HA antibody, we added 0.1 U/mL exogeneous sialidase from *Clostridium perfringens* (*Cp*NA). Although this treatment is sufficient to restore viral shedding in the presence of the potent NA inhibitor oseltamivir carboxylate (Fig. 2I) and has previously been used to rescue viruses completely lacking NA (25), it did not restore viral release in the presence of any of the stalk-binding antibodies tested, suggesting that these antibodies are able to restrict viral release through mechanisms other than inhibition of NA.

While FluA-20 IgG and FISW84 IgG both bound to cells infected by A/WSN/1933 (Fig. S2A), they did not reach 50% reduction in virion shedding at the maximum concentration tested (Fig. 2E and F). We reasoned that this may be due to limited accessibility of the epitopes these antibodies recognize. The FISW84 epitope likely requires tilting of the HA ectodomain to enable binding (20, 21), and the FluA-20 epitope is occluded in the HA trimer, requiring transient opening of the HA head for this antibody to bind. FISW84 IgG inhibited neither A/WSN/1933 nor a virus with HA from A/California/04/2009 (Fig. S2B) although it remains to be determined if this result generalizes to other antibodies targeting the anchor epitope. In contrast, we observed significant inhibition of viral shedding by FluA-20 against the A/California/04/2009 reassortant viruses, whose HA readily dissociates into monomers (Fig. S2C) (26). The increased potency of FluA-20 against HA from A/California/04/2009 versus HA from A/WSN/1933—despite the conservation of the five residues with which FluA-20 primarily interacts (18, 19)—highlights the importance of HA trimer stability and epitope accessibility in determining antibody potency in inhibiting virus release.

### Inhibition of viral egress by anti-HA antibodies affects the morphology of released virions

To examine the effect of antibody inhibition on the characteristics of released viruses, we compared particles from the filamentous strain A/Hong Kong/1968 raised in the presence or absence of CR8020 IgG. At antibody concentrations where particle release is decreased by 75%, we observe a 36% apparent decrease in mean HA abundance per particle, measured using a fluorescent Fab fragment from C05 (Fig. S1C). This decrease in HA intensity may result from changes in HA abundance per particle or from interference of CR8020 IgG with C05 Fab attachment. To compare particle size distributions in a way that is independent of Fab labeling intensity, we measured the percentage of viral filaments greater than 1, 2, or 4 µm in length, sizes above the diffraction limit of our optical system (~300 nm) which can easily be resolved. We find that the percentage of viral filaments above each length threshold successively decreases in the presence of CR8020 IgG relative to the antibody-free condition, suggesting that antibodies can alter both the number and the morphological features of the viruses that are released over the course of infection (Fig. S1D).

### Anti-NA antibodies inhibit viral release via mechanisms beyond direct inhibition of enzymatic activity

**Fig 3:**
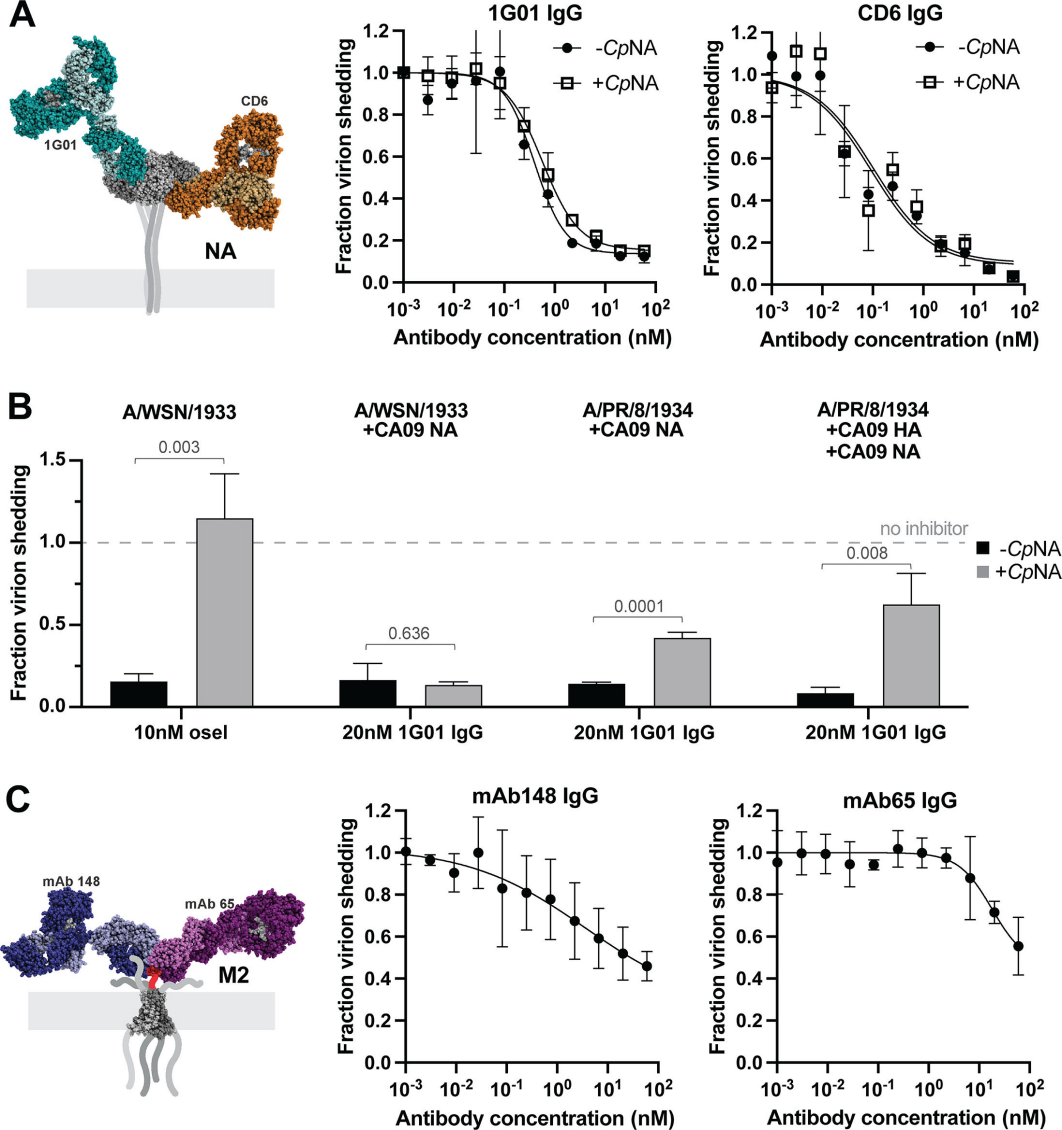
Anti-NA and anti-M2 antibodies inhibit influenza virus release. (**A**) Neutralization curves for the anti-NA antibodies 1G01 and CD6 IgG against A/WSN/1933 with NA from A/California/04/2009. Experiments are performed with or without 0.1 U/mL exogenous sialidase (“*Cp*NA”). The models of antibodies bound to NA are obtained from PDB IDs 6Q23 and 4QNP. Curves are generated from three biological replicates. (**B**) Data showing the effect of NA inhibition by oseltamivir or 1G01 in the presence or absence of exogenous sialidase (*Cp*NA) across different genetic backgrounds. Data for oseltamivir with or without *Cp*NA are repeated from Fig. 2I using the A/WSN/1933 strain. Data are from three biological replicates. *P* values are determined by independent *t*-tests. (**C**) Measurement of anti-M2 antibodies mAb148 and mAb65 against A/WSN/1933. The models of antibodies bound to the M2 ectodomain are obtained from PDB IDs 2L0J, 4N8C, and 5DLM. Error bars in A–C show standard deviations; fit curves in A and C are generated by the least squares method.

We next tested two anti-NA antibodies: 1G01, which binds to the active site (9), and CD6 which binds to the interface of adjacent monomers in the NA tetramer (27). Since both antibodies inhibit NA enzymatic activity, we performed these experiments in both the presence and absence of *Cp*NA, which has been shown by us (Fig. 2I) and by others to compensate for loss of NA enzymatic activity (25, 28). We found that the extent to which inhibition of viral shedding by 1G01 and CD6 IgG could be rescued by *Cp*NA varied depending on the virus’ genetic background. Although all viruses tested express the same NA (from A/California/04/2009), particle release could not be rescued by *Cp*NA in a WSN33 background with mismatched HA (Fig. 3A) but was partially restored in a PR8 background with matched or mismatched HA (Fig. 3B). This suggests that anti-NA antibodies inhibit viral release through mechanisms besides blocking enzymatic activity, and that this inhibition varies depending on the genetic context.

### Anti-M2 antibodies moderately reduce viral release at high concentrations

Finally, we investigated the ability of two M2-specific IgG antibodies, mAb148 (29)and mAb65 (30), to inhibit virus assembly and release. These antibodies bind to overlapping epitopes in the extracellular domain of M2. While prior work found that an antibody against the M2 ectodomain (14C2) only inhibited the assembly of filamentous strains (8, 31), both anti-M2 antibodies we tested were able to restrict viral shedding of the spherical strain WSN33 but required high concentrations and were generally less potent than HA- and NA-specific antibodies (Fig. 3C). Together with our results testing anti-HA and anti-NA antibodies, this establishes inhibition of viral release as a widespread mechanism of protection for antibodies targeting each of the three primary viral surface proteins.

### Crosslinking of HA or NA in *cis* or in *trans* contributes to inhibition of viral release

**Fig 4:**
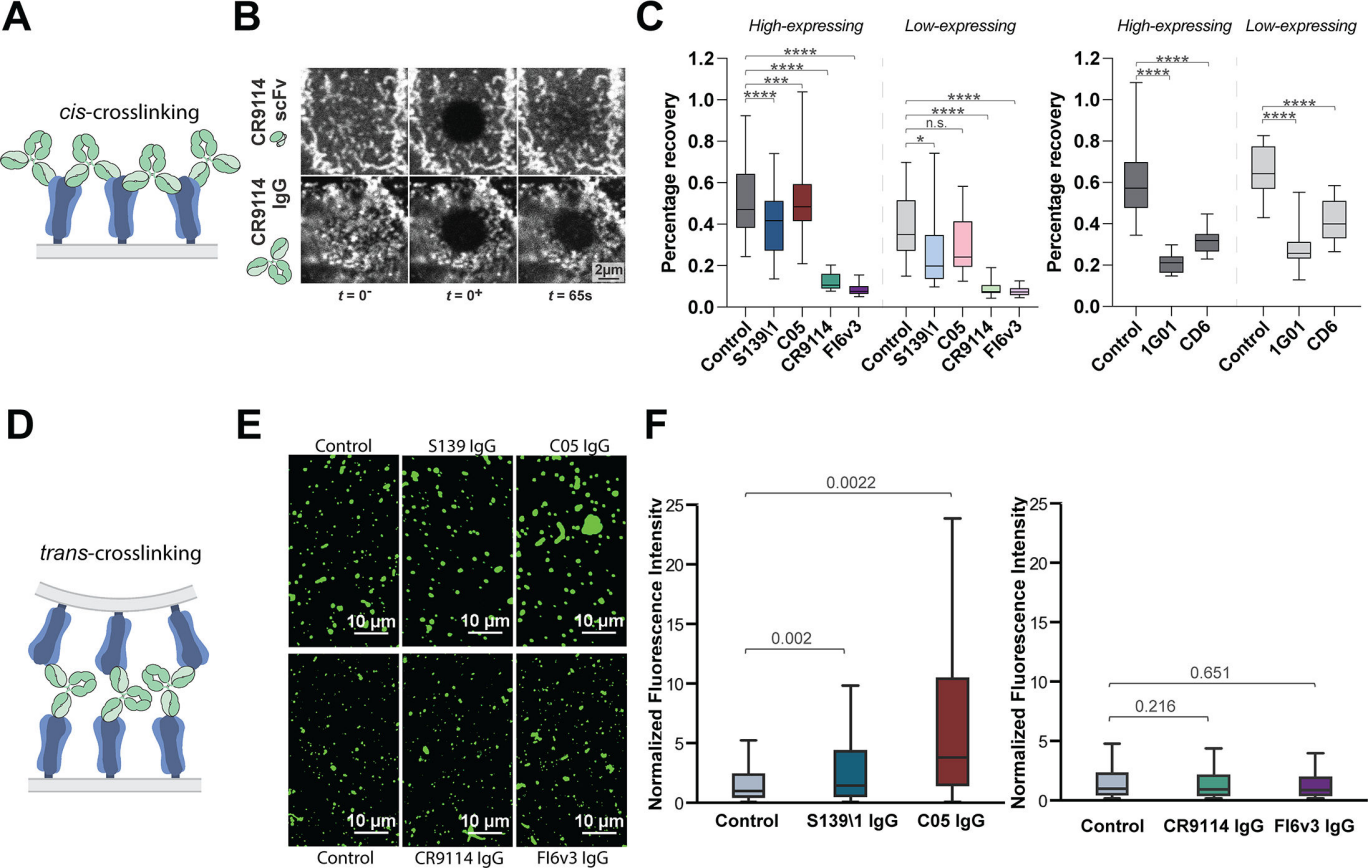
Antibodies inhibit virus assembly and release through distinct mechanisms. (**A**) Cartoon illustration of *cis* crosslinking of HA by bivalent IgG antibodies. (**B**) Sample images of the apical cell surface immediately before and after photobleaching (*t* = 0 and 0^+^) and at 65 s afterwards. (C, left) Percentage recovery of photobleached HA at 65 s post-photobleaching, as shown in panel B. Antibodies are tested at their respective IC75_release_ values. Data are combined from at least 50 photobleached cells per condition. Analyzed cells are split into high- and low-expressing groups based on the intensity of HA staining on the cell surface. (C, right) Percentage recovery of photobleached NA at 65 s post-photobleaching. Data are combined from 30 photobleached cells per condition. *P* values are determined by Mann-Whitney tests (* indicates *P* < 0.05, ** indicates *P* < 0.01, *** indicates *P* < 0.001, **** indicates *P* < 0.0001, and n.s. indicates not significant). (**D**) Cartoon illustration of *trans* crosslinking of HA by bivalent antibodies. (**E**) Sample images of virus particles/aggregates. (**F**) Distributions of particle/aggregate size, measured via fluorescence intensity. Antibodies are tested at their respective IC75_release_ values. Fluorescence intensities are normalized to the median value of their respective control groups. Results from RBS-binding and stalk-binding antibodies are plotted separately because different non-competing fluorescent Fabs are used to measure particle/aggregate size. Data are combined from three biological replicates using stocks of A/WSN/1933 expanded separately. *P* values are determined using the mean of individual biological replicates by paired *t*-tests.

To understand mechanisms that contribute to antibody inhibition of viral release, we compared inhibition profiles of bivalent CR9114 IgG and monovalent CR9114 Fab at concentrations up to ~100-fold higher than the dissociation constant for both Fab and IgG (15). In contrast to CR9114 IgG, the monovalent CR9114 Fab showed no inhibition of viral release, confirming that bivalency is important. Together with our finding that exogenous sialidase has limited ability to reverse inhibition of viral egress by anti-NA antibodies but not oseltamivir, this suggests that antigen crosslinking plays a key role in inhibition of viral shedding. This crosslinking could occur between antigens within the same membrane (i.e., in *cis*; Fig. 4A) or between antigens in closely apposed membranes (i.e., in *trans*). Although antigen crosslinking has been observed for some influenza-specific antibodies (14, 15, 32–35), it remains unclear how common this phenomenon is and how it depends on the specific epitopes which an antibody binds to.

*Cis* crosslinking of trimeric or tetrameric viral surface proteins by bivalent antibodies could result in extensive networks of proteins with reduced mobility, a scenario that can be readily detected using fluorescence recovery after photobleaching (FRAP) (36). To avoid changes in protein mobility that could arise in the context of productive infection, we performed FRAP on cells transfected with either HA or NA plasmids and treated with bivalent or monovalent targeting antibodies at ~48 hours post-transfection (Fig. 4B). While HA bound by monovalent CR9114 scFv showed efficient recovery (60% after 65 s), HA bound by bivalent CR9114 or FI6v3 IgG at concentrations that inhibit 75% of viral release did not significantly recover (Fig. 4C, left). In comparison, RBS-binding antibodies S139\1 and C05 IgG showed only modest *cis* crosslinking, regardless of HA expression level (Fig. 4C, left). Similar experiments evaluating NA mobility in the presence or absence of 1G01 or CD6 IgG demonstrate that both antibodies significantly reduce NA diffusion, consistent with *cis* crosslinking (Fig. 4C, right).

We next investigated the ability of antibodies to crosslink HA across membranes in *trans* (Fig. 4D). Previous studies have shown that RBS-specific antibodies can cause virus aggregation on infected cell surfaces or in suspension (14, 32–34). To compare *trans* crosslinking across the antibodies in our panel, we incubated virus particles overnight with antibodies at concentrations that result in 75% inhibition of virus release and measured particle aggregation using fluorescence microscopy (Fig. 4E). While C05 and S139\1 IgG resulted in significant aggregation relative to IgG-free controls, the stalk-binding antibodies CR9114 and FI6v3 IgG did not (Fig. 4F). These observations suggest that membrane-distal epitopes support antigen crosslinking across membranes, while membrane-proximal epitopes restrict crosslinking to antigens within the same membrane.

### Antibodies have widely varying potencies in inhibiting viral entry and release

**Fig 5:**
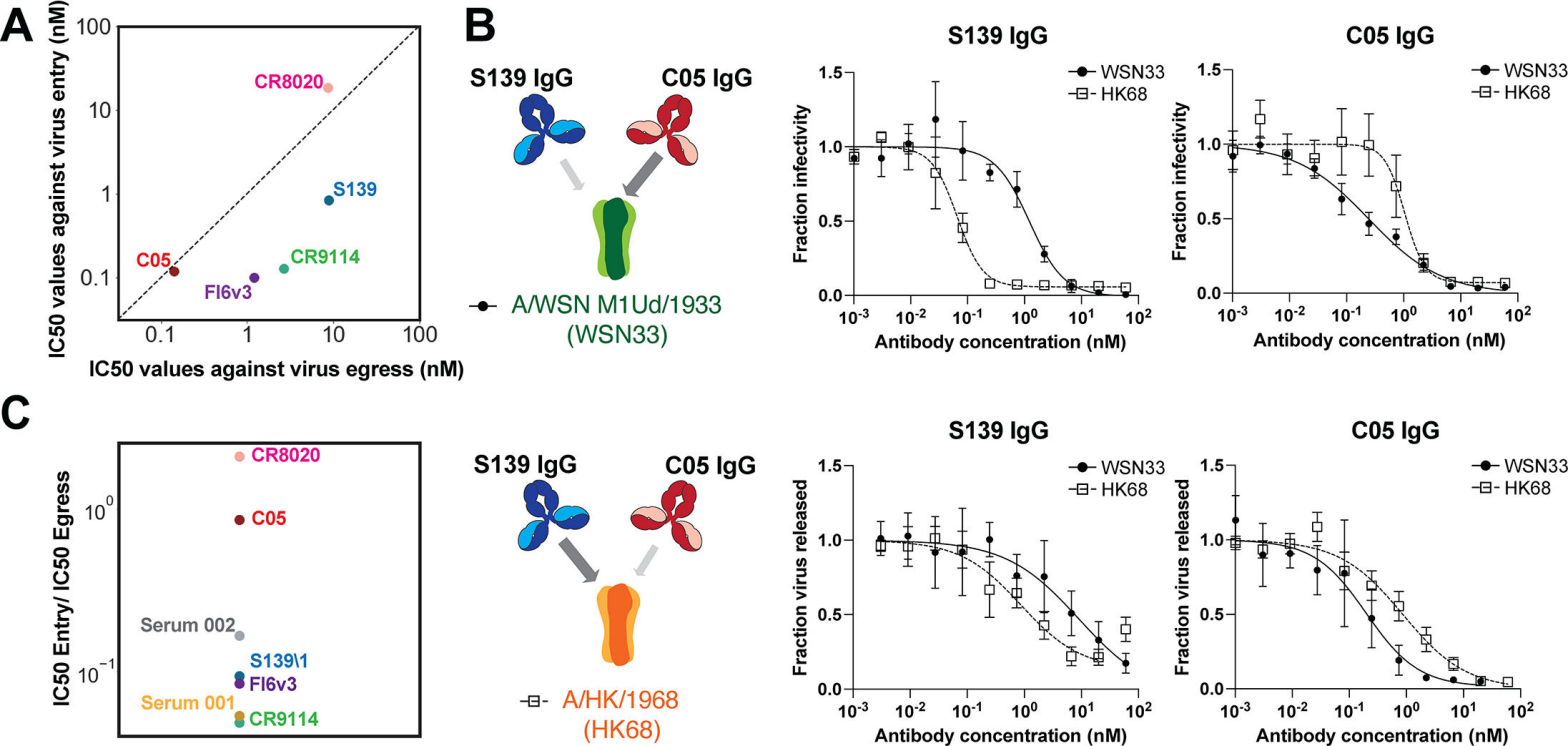
Antibodies and human sera differ widely in their ability to inhibit viral entry and viral egress. (**A**) Plot showing IC50 values for antibody inhibition of viral release and entry for the classically neutralizing antibodies from Fig. 2. (B, Left) Illustration of binding preferences of S139\1 and C05 for different HAs. Wider arrows indicate stronger binding. (B, top right) Neutralization curves for S139\1 and C05 against viral entry. (B, bottom right) Neutralization curves for S139\1 and C05 against viral release. (**C**) Ratio of IC50_entry_ to IC50_release_ for antibodies and human convalescent sera. Data in panels B and C are from three biological replicates.

Many of the anti-HA antibodies from our panel have established functions in blocking viral entry, by preventing either attachment (S139\1, C05) or membrane fusion (CR9114, FI6v3, and CR8020). We sought to determine how the potency of these antibodies at inhibiting viral entry (“IC50_entry_,” measured using single-round microneutralization assays) compares to their potency at inhibiting viral release (“IC50_release_”). It is worth noting that apparent values for IC50_release_ may underestimate antibody potency in some cases, since increased expression of viral antigens over time will reduce the concentration of antibody in solution relative to the initial value; thus, IC50_release_ is best interpreted as a practical estimate of the inhibitory concentration as opposed to a true but theoretical value. We find that the ratio of IC50 values varies widely across antibodies (Fig. 5A). For example, although S139\1 and C05 both inhibit viral attachment, the two antibodies differ ~10-fold in their IC50 ratio: specifically, C05 is similarly potent in inhibiting viral entry and release while S139\1 is ~10-fold more effective at blocking viral entry than viral release. When we tested these antibodies against HAs toward which they have different affinities (HK68 and WSN33), we found that the ratio between IC50_entry_ and IC50_release_ remained similar for both antibodies (Fig. 5B; Table S1). Thus, while the absolute potency of an antibody at inhibiting viral release depends on its affinity, its relative potency at inhibiting entry versus release appears to depend on other factors. Interestingly, inhibition of virus shedding by S139\1 IgG decreases at antibody concentrations above ~5 nM. This is consistent with a transition from bivalent to monovalent binding as antibodies in solution begin to compete with bound antibodies for free HAs, disrupting *cis* or *trans* crosslinking (37). This phenomenon may limit inhibition of viral release for antibodies with exceptional affinity.

To investigate how results using monoclonal antibodies compare with polyclonal mixtures, we measured inhibition of entry and release by human convalescent sera with A/California/04/2009. Across two samples, we observe that one (Serum 1) is ~20-fold more potent at inhibiting viral entry relative to viral release, while the other (Serum 2) is ~6-fold more potent (Fig. 5C). This is broadly consistent with our experiments with monoclonal antibodies and indicates that inhibition of viral entry does not necessarily predict potency in inhibiting viral release. Collectively, these data demonstrate that antibody inhibition of viral release plays a supporting role in limiting the spread of infection by a wide variety of antibodies, occasionally rivaling inhibition of viral entry even for antibodies that block receptor binding or membrane fusion.

### Antibody-HA structures predict a broad range of *cis* and *trans* crosslinking preferences

**Fig 6:**
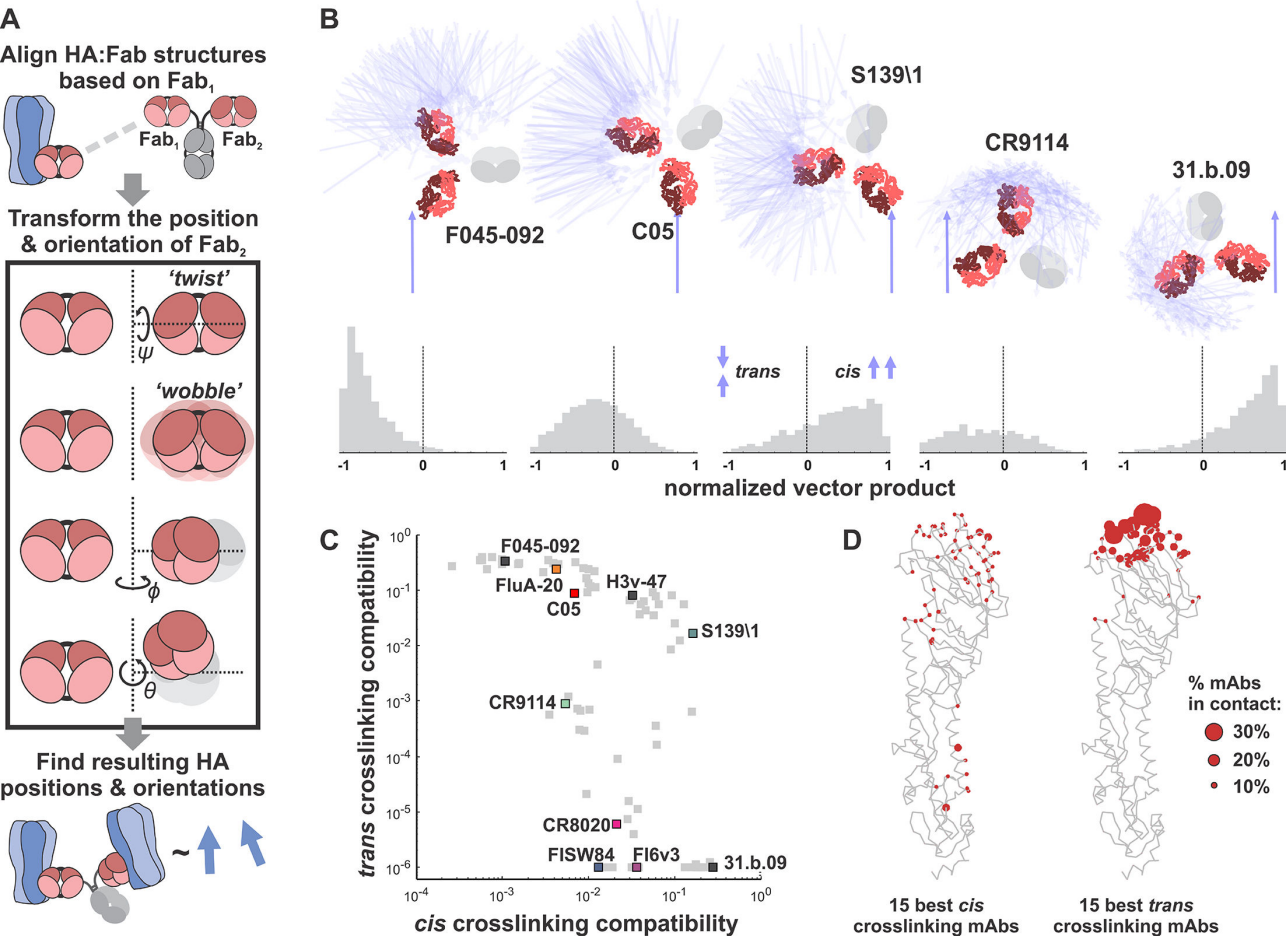
A structure-based model predicts *cis* and *trans* crosslinking preferences for anti-HA antibodies. (**A**) Schematic overview of the modeling approach illustrating the transformations used to sample potential configurations of the two antibody Fab fragments (Fab_1_ and Fab_2_) and the corresponding positions and orientations of the bound HAs. (**B**) Simulation results for selected antibodies. Top: bound HAs are represented as blue vectors, Fab fragments are shown in red, and the antibody Fc (not included in the simulation) is shown in gray. Bottom: distributions of relative HA orientations for the depicted antibodies. *Trans* crosslinking corresponds to anti-parallel HA orientation while *cis* crosslinking corresponds to parallel HA orientation. The resulting HA distributions are subsequently filtered to remove sterically forbidden configurations (Fig. S3A). (**C**) Predicted *cis* and *trans* crosslinking compatibilities for Fab:HA structures. Select antibodies characterized in this work or otherwise of note (F045-092, H3v-47) are highlighted. (**D**) Distribution of contact sites for the 15 best *cis* crosslinking (left) and *trans* crosslinking (right) antibodies based on the analysis shown in panel C. The backbone of an HA monomer is shown in gray. The size of the red spheres indicates the percentage of top antibodies that contact particular residues.

We reasoned that the binding orientation of an antibody could influence its geometric preference for crosslinking in *cis* or in *trans*. To investigate this possibility, we developed a geometric model using structures of Fab fragments bound to HAs of different subtypes. Using each structure to constrain the position for one of the antibody’s Fab arms, the model randomly samples potential configurations for the second Fab arm and evaluates the compatibility of each sampled configuration with *cis* or *trans* crosslinking (Materials and Methods, Fig. 6A and B; Fig. S3A and B). This model focuses on the effects of antibody binding position and orientation without accounting for affinity, kinetics, or epitope accessibility—factors which are also likely important. Among the antibodies we tested, predictions from this model qualitatively agree with experimental measurements. C05 has a high propensity for *trans* crosslinking, while for the stalk-binding antibodies CR9114, FI6v3, and CR8020, *cis* crosslinking is preferred (Fig. 6B and C). Also consistent with our crosslinking data (Fig. 4), S139\1 is predicted to have a reasonable propensity for both crosslinking modes.

Among antibodies that we have not tested, F045-092 (38), L3A-44 (39), and CH65 (40)—antibodies which all bind in or around the RBS—are predicted to have a strong preference for *trans* crosslinking (Table S2). In contrast, antibodies predicted to have a strong preference for *cis* crosslinking bind to a range of antigenic sites, including adjacent to the RBS [S139\1 (12)], the central stalk [31.b.09 (41)], and the trimer interface [S8V2-37 (42)] (Fig. 6C and D; Table S2). We selected one of these antibodies, 31.b.09, for further testing. Although 31.b.09 binds to an epitope that largely overlaps that of CR9114, the heavy and light chains are rotated ~180^o^ in the structures of these Fabs bound to the HA central stalk (Fig. S3C); positioning 31.b.09 in a way that the models predict would promote *cis* crosslinking and perhaps make it a relatively better inhibitor of virus assembly. While CR9114 inhibits viral entry ~20-fold more potently than virus assembly and release, 31.b.09 shows the opposite trend, inhibiting assembly and release more effectively than viral entry (Fig. S3D). Collectively, the general agreement between structure-based predictions and our experimental results suggests that antibody binding orientation constrains crosslinking propensity and may provide a metric for predicting inhibition of viral assembly and release. However, we note that additional parameters not included in the model, such as binding kinetics, are likely necessary to predict the trends observed in our experimental data (Fig. 5A and C).

## DISCUSSION

While antibody inhibition of virus release has been documented in the context of infection by influenza and other enveloped viruses (43, 44), it remains an understudied aspect of antibody function. For the most part, antibodies that inhibit viral release have been identified via a process of elimination, through their inability to block viral attachment or entry despite restricting viral replication *in vitro*. While traditional assays such as western blotting and hemagglutination can be used to estimate the number of viral particles released during infection, the approach described here is comparatively direct and insensitive to the antibody that is being tested. By systematically and quantitatively comparing inhibition of virus release by a variety of neutralizing antibodies targeting distinct antigenic sites on HA, NA, and M2, we establish the generality of this phenomenon and identify distinct mechanisms through which antibodies restrict viral shedding: by crosslinking viral surface proteins in *cis*, to disrupt the diffusion of viral surface proteins or by crosslinking *in trans*, to induce viral aggregation or sequestration on the cell surface. In addition to reducing the number of virus particles released during infection, we find that antibodies can also alter the size or morphology of released particles, potentially influencing viral replication in other ways. While we have focused specifically on IgG1 antibodies, other isotypes abundant in mucosal environments—especially IgA and IgM—will likely increase *trans*-crosslinking potency and may extend this capability to antibodies that recognize membrane-proximal epitopes. We propose that inhibition of virus assembly may serve as an additional metric to evaluate antibody potency alongside traditional neutralization measurements and assays aimed at evaluating Fc-dependent effector functions.

While the neutralizing activities of antibodies during viral entry are often well correlated with their binding affinity, inhibition of assembly and release appears to depend on additional factors. One factor identified here is the antibody binding geometry. We find that the ability of antibodies to bridge two antigens—either in the same membrane (*cis*) or in opposing membranes (*trans*)—can be predicted with reasonable accuracy using a simplified computational model. This presents an opportunity to identify candidate antibodies that bind to neutralizing epitopes in favorable orientations that maximize crosslinking. We would expect that such antibodies would have dual potency in preventing viral entry as well as egress, given that their affinity is sufficiently high. A second factor that can contribute to inhibition of viral release is functional interactions between the target protein and other viral proteins; in particular, we find that the potency and mechanism with which anti-NA antibodies inhibit viral release depends on the HA and other viral proteins with which NA is paired. This result parallels early observations with anti-M2 antibodies, in which the potency of the ectodomain-specific antibody 14C2 was observed to differ markedly between strains despite complete conservation of the antibody epitope (8, 31). Understanding the polygenic nature of antibody inhibition of IAV release will require a deeper understanding of the mechanisms through which the IAV surface proteins contribute to assembly and budding.

We expect that many of the considerations that make an antibody a potent inhibitor of IAV assembly will also apply to other viruses that assemble at the plasma membrane of infected cells. Previous studies have reported that broadly neutralizing antibodies against E1 and E2 glycoproteins on alphaviruses are able to inhibit viral egress (45–50). While crosslinking likely plays a role in this process, the ability of some E1-specific Fab fragments to inhibit alphavirus release similar to their IgG counterparts suggests that crosslinking is not a requirement (48). More generally, we speculate that viruses whose membrane proteins directly interact with each other on the viral surface or during assembly (51–53) may be vulnerable to inhibition by non-crosslinking antibodies that disrupt these interactions. Further investigation into the mechanisms through which antibodies restrict the assembly and release of influenza and other viruses may help guide the development of more broadly protective therapies and vaccines.

## MATERIALS AND METHODS

### Cell lines and viruses

MDCK-II and HEK-293T cell lines used in the study were purchased as authenticated cell lines (STR profiling) from ATCC and cultured with cell growth medium comprised of Dulbecco’s modified Eagle’s medium (DMEM; Gibco) supplemented with 10% fetal bovine serum (FBS; Gibco) and 1× antibiotic-antimycotic (Corning) under standard conditions (37°C and 5% CO_2_).

Recombinant viruses were rescued using standard reverse genetics techniques (54). In brief, co-cultures of HEK-293T and MDCK-II were transfected with plasmids containing each vRNA segment flanked by bidirectional promoters. Viruses were collected from co-cultures at around 2 days post-transfection and plaque purified. Viral plaques were passaged at an MOI of ~0.001 in MDCK-II cells in virus growth medium comprised of Opti-MEM (Gibco), 2.5 mg/mL bovine serum albumin (Sigma-Aldrich), 1 µg/mL L-(tosylamido-2-phenyl ethyl) chloromethyl ketone (TPCK)-treated trypsin (Thermo Scientific Pierce), and 1× antibiotic-antimycotic (Corning). The viral stocks were further expanded by passaging at low MOI.

To obtain A/WSN/1933 viruses with filamentous phenotype, the M1 sequence within the WSN M segment is replaced by that of A/Udorn/1972. Rescue and characterization of these viruses have been described previously (55). To obtain A/California/2009 reassortant viruses, HA and/or NA segments from A/California/2009 are transfected in combination with corresponding genetic segments from A/WSN/1933 or A/Puerto Rico/8/1934.

### Antibody purification and labeling

Sequences for the variable regions of antibody heavy and light chains were obtained from deposited antibody structures on the PDB and cloned into expression vectors to make full-length human IgG1 antibodies. Sequences for the heavy chain were modified with a C-terminal ybbR tag for enzymatic labeling (56) and, in the case of Fab fragments, a His6 tag for affinity purification using Ni-NTA Agarose Beads (Thermo Scientific Pierce). Full-length antibodies were purified using protein A agarose beads (Thermo Scientific Pierce). Antibodies were expressed in HEK-293T following transfection with heavy and light chains at >70% confluency. Cells were subsequently cultured for 7 days in Opti-MEM with 1× Anti-Anti and with or without 2% FBS for Fab and IgG antibodies, respectively. Supernatants from the HEK-293T cells were collected for affinity purification. Full details on antibody purification and enzymatic labeling are described elsewhere (57).

Human convalescent sera were obtained through BEI Resources (NR-18964 and NR-18965 for Serum 001 and Serum 002, respectively) and used without further purification in virus counting assays and microneutralization assays. IC50 values for serum neutralization were measured as fold dilutions from the initial undiluted stock.

### Virus counting assay

MDCK-II cells were seeded into 96-well plates as a monolayer. Cells were washed twice with PBS and infected at MOI ~1 for 2 hours at 37°C. At 2 hpi, the inoculum was removed, and cells were washed vigorously with PBS twice. Antibodies were serially diluted in virus growth media and added to cells for an additional 6 hours at 37°C. Unless otherwise indicated, media were supplemented with 0.1 U/mL *Cp*NA (Sigma-Aldrich) to minimize effects from steric inhibition of NA by stalk-binding antibodies. At 8 hpi, the supernatant was collected, diluted to the appropriate viral concentration to assure linear quantification (Fig. 1B), and mixed with site-specifically labeled viruses with sulfo-Cy5 conjugated to HA [prepared as previously described (55)]. Sulfo-Cy5 labeled virus served as a loading control to improve the consistency of quantification. Fluorescent anti-HA single-chain variable fragments (scFvs) or Fabs that recognize non-competing epitopes against the antibodies under test are used to label sample virus and the sulfo-Cy5 labeled loading control. For A/WSN/1933 challenged with HA head-specific mAbs (S139\1, C05), we labeled collected viruses with fluorescent CR9114 scFv. For A/Hong Kong/1968 challenged with HA head-specific mAbs, we labeled collected viruses with fluorescent FI6v3 scFv. For A/WSN/1933 and A/Hong Kong/1968 challenged with HA-stalk-specific antibodies, we labeled collected viruses with fluorescent C05 Fab. For A/California/04/2009 reassortant viruses challenged with fluorescent FISW84 IgG, we labeled collected viruses with CR9114 scFv in the same wavelength to avoid omitting particles, since the FISW84 and CR9114 binding footprints partially overlap. All samples were imaged with a Nikon Ti2 confocal microscopy system using a 40×, 1.3-NA objective. Images containing virus particles were then analyzed using spot detection in each channel to determine the total number of virions immobilized on the glass-bottom well. The ratio between the particle count in the experimental and control samples was calculated for each antibody concentration and normalized to the antibody-free condition to generate the neutralization curve.

Imaging plates for virus quantification were prepared by coating coverslip bottom wells (Cellvis) with 0.18 mg/mL BSA-biotin in PBS overnight at 4°C. The imaging plate was then washed with PBS twice and incubated with 25 µg/mL streptavidin (Invitrogen) in PBS for 2 hours. The imaging plate was then washed twice with PBS and incubated with 25 µg/mL biotinylated *Erythrina cristagalli* lectin (Vector Laboratories) at room temperature for 2 hours. Finally, the imaging plate was washed twice with NTC buffer (100 mM NaCl, 5 mM CaCl_2_; 20 mM Tris pH 7.4) prior to adding virus samples.

As a comparison to the virus counting assay, we performed quantitative western blotting using the same serially diluted input virus stock used to generate the assay validation curve (Fig. 1C). The same volume of virus samples was used as input for the imaging assay and for western blotting. The blot was incubated with a polyclonal anti-HA primary antibody (Invitrogen PA5-34929) overnight at 4°C and probed with an IRDye 800CW goat anti-rabbit secondary (Licor) at room temperature for 1 hour prior to scanning on a Licor Odyssey imager.

### Microneutralization assay

MDCK-II cells were seeded into 96-well plates as a monolayer. Monoclonal antibodies were serially diluted and mixed with viruses at 4°C for 2 hours. Cells were washed twice with PBS prior to adding the antibody-virus mixture and incubated at 37°C. At 2 hours, the antibody-virus mixture was removed, and cells were washed twice with PBS and replenished with virus growth media containing 0.125 U/mL *Cp*NA to prevent delayed primary infection from residual virus. The cells were incubated at 37°C for 6 hours and imaged using CR9114 scFv 488 or FI6V3 scFv 488 to label HA on the surface of infected cells. The number of HA-positive cells were counted using the Spot Detection function in the Nikon Elements Analysis software and normalized to the antibody-free condition to generate the neutralization curve.

### Virus aggregation assay

Freshly expanded A/WSN/1933 viruses were treated with 0.125 U/mL *Cp*NA for 2 hours at 4°C. Viruses were then incubated overnight at 4°C with antibodies at their respective IC75 values for inhibition of virus release (Fig. 2). Virus-antibody complexes were then immobilized via ECL onto glass-bottom imaging chambers and labeled with fluorescent non-competing CR9114 scFv or C05 Fab and imaged using a 40×, 1.30-NA objective. Images of virus-antibody complexes were then segmented using Nikon Elements Software, and the HA intensity from the fluorescent non-competing Fab or scFv was quantified. Only viruses visualized with the same antibody fragments are directly compared in Fig. 4F.

### Fluorescence recovery after photobleaching assay

An 8-well-chambered cover glass (Cellvis) was incubated with 10 µg/mL Human Plasma Fibronectin (EMD Millipore) at 4°C for 20 minutes. HEK-293T cells were transfected with HA- or NA-expressing plasmids and split into the imaging chambers. At ~48 hours post-transfection, fluorescently labeled antibodies are added to each well at the measured IC75 value (Fig. 2 and 3) to perform FRAP. CR9114 scFv and CD6 Fab are used as negative controls to measure the normal diffusion of HA and NA in the absence of bivalent antibodies. Due to the slow dissociation rates of CR9114 scFv and CD6 Fab to WSN HA and CA NA, respectively, the FRAP measurement is not complicated by rapid antibody turnover. An Olympus FluoView FV1200 laser scanning confocal microscope with a 60×, 1.35-NA objective was used for photobleaching and image acquisition. Photobleaching was performed over a circular region 1.98 µm in diameter using maximum laser power. One frame was taken before bleaching, and one frame was taken 1 s after the bleaching event. Fourteen frames in total were collected at 5-s intervals following the photobleaching event.

### Measuring virus particle size

Images of shed virus particles collected with Nikon TI2 confocal microscopy system using a 60×, 1.40-NA objective were segmented and quantified to determine the major and minor axis length and HA intensity measured by fluorescent non-competing antibodies as previously described (55).

### Modeling antibody crosslinking from structural information

To model the propensity of different mAbs to crosslink HA in *cis* or in *trans* based on their binding orientation, atomic coordinates for 83 structures of non-duplicate Fab fragments bound to HAs of different subtypes were downloaded from the Protein Data Bank and aligned by the Fab heavy and light chains. Using this first Fab arm (“Fab_1_”) as a reference, a range of possible configurations for the alternate Fab arm (“Fab_2_”) was sampled through sequential translation and rotation transformations, including introducing symmetry-breaking “wobble” in Fab_2_, as observed for IgG antibodies (58–60) (Fig. 6A). Based on previous estimates from electron microscopy, Fab_2_ conformations using *ψ* = ±60°, *ϕ* = ±30°, and *θ* = 60° ±30° were sampled, where “±” indicates the standard deviations of the distribution each angle is sampled from. These simplified parameters result in a Fab arm which can twist extensively about its major axis (61) and which can rotate extensively along the other principal axes as well, consistent with previous observations (58–60).

Each sampled configuration of Fab_1_ and Fab_2_ results in a relative position and orientation for the bound HAs which was evaluated for its compatibility with *cis* or *trans* crosslinking. Configurations where the stalk-to-stalk or head-to-head distances are less than 5 nm are eliminated, and head-to-stalk distances less than 10 nm are eliminated as well (Fig. S3A). For *trans* crosslinking, the head of the second (inverted) HA was required to be above the head of the first (upright) HA, while for *cis* crosslinking, the head of the second HA was required to be above the base of the first HA. For each allowable configuration, the dot product of axial vectors that extend from the bottom to top of the two HAs was evaluated. This produced a distribution of values ranging from −1 (for anti-parallel HAs) to +1 (for parallel HAs) which was used to score the geometric propensity of a given mAb to crosslink HAs in *cis* and in *trans*; antibodies whose distribution of vector products is clustered at +1 are highly compatible with *cis* crosslinking, while antibodies where the distribution clusters around −1 are highly compatible with *trans* crosslinking (Fig. 6B). Changing the range of angular distributions sampled to determine possible configurations for bound HAs influences the predictions quantitatively but not qualitatively. Fig. S3B shows how the predicted *cis* and *trans* crosslinking propensities for each mAb change under different implicit flexibilities; the dominant effect is to increase the ability of most antibodies to crosslink in *cis*, with little predicted effect on their ability to crosslink in *trans*.

### Statistics, replicates, and software

Statistical analysis was performed using GraphPad Prism 9, MATLAB, or Python Scipy 1.7.3. No statistical methods were applied to predetermine sample size. Statistical tests used are indicated in each respective figure legend. Box plots may omit outliers that are beyond the limit of the y-axes for clear visualization, but these are included in statistical analyses. Fit curves are generated by the least squares method using GraphPad Prism 9. Biological replicates are defined as cells separately infected/transfected/treated and assayed as indicated.

## Data Availability

Analyzed data are available in the article. Raw images are available at Open Science Framework. Analysis code and code used for Fig. 6 and Fig. S3A and B are available at GitHub.

## SUPPLEMENTAL MATERIAL

Fig. S1 jvi.01398-23-s0001.tif Virus morphology does not strongly affect inhibition of viral egress, but neutralizing antibodies can alter the apparent size of released virions.

Fig. S2 jvi.01398-23-s0002.tif Binding and neutralization of antibodies with limited epitope access.

Fig. S3 jvi.01398-23-s0003.tif Predicting *cis* and *trans* crosslinking with a structure-based model.

Supplemental legends and Table S1 jvi.01398-23-s0004.docx Legends for supplemental figures. Table S1. Inhibition of viral entry and release for S139\1 and C05 against different viral strains.

Table S2 jvi.01398-23-s0005.xlsx Predicted *cis* and *trans* crosslinking propensities from HA-Fab structures.

ASM does not own the copyrights to Supplemental Material that may be linked to, or accessed through, an article. The authors have granted ASM a non-exclusive, world-wide license to publish the Supplemental Material files. Please contact the corresponding author directly for reuse.
