# Supplementary figures and images for: Antibody inhibition of influenza A virus assembly and release
Source: J Virol. 2024 Jan 5;98(2):e01398-23. doi: 10.1128/jvi.01398-23 (PMC10878280; doi:10.1128/jvi.01398-23)

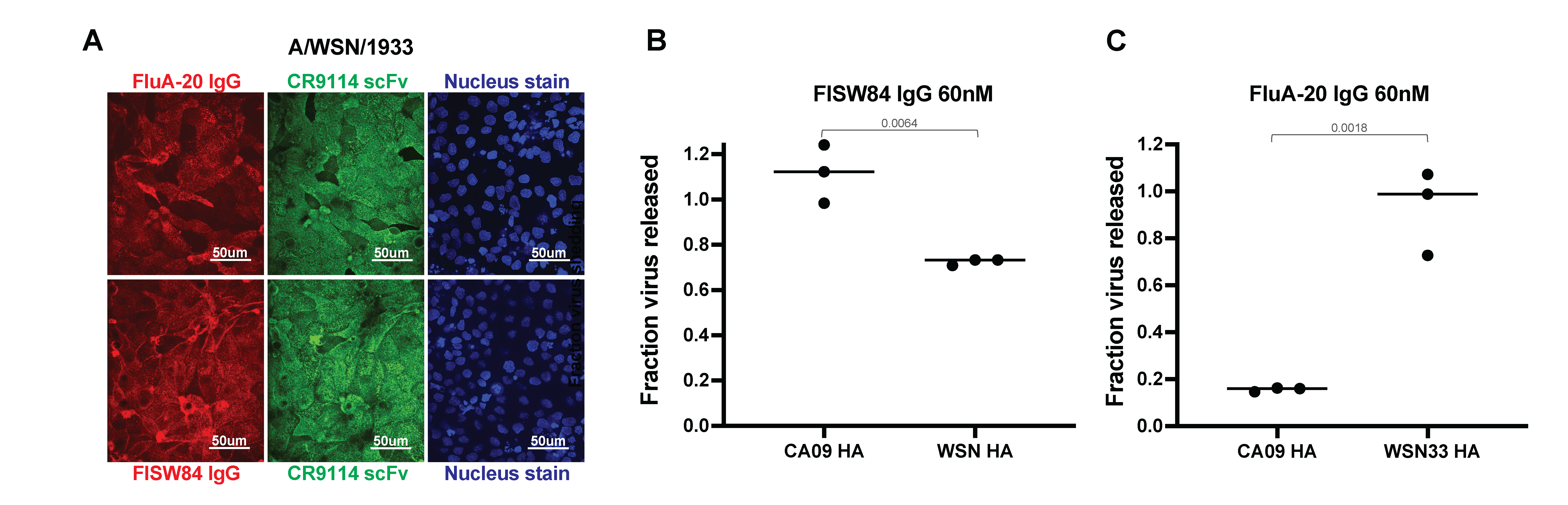

Supplement: Fig. S2 — Binding and neutralization of antibodies with limited epitope access. [file jvi.01398-23-s0002.tif]

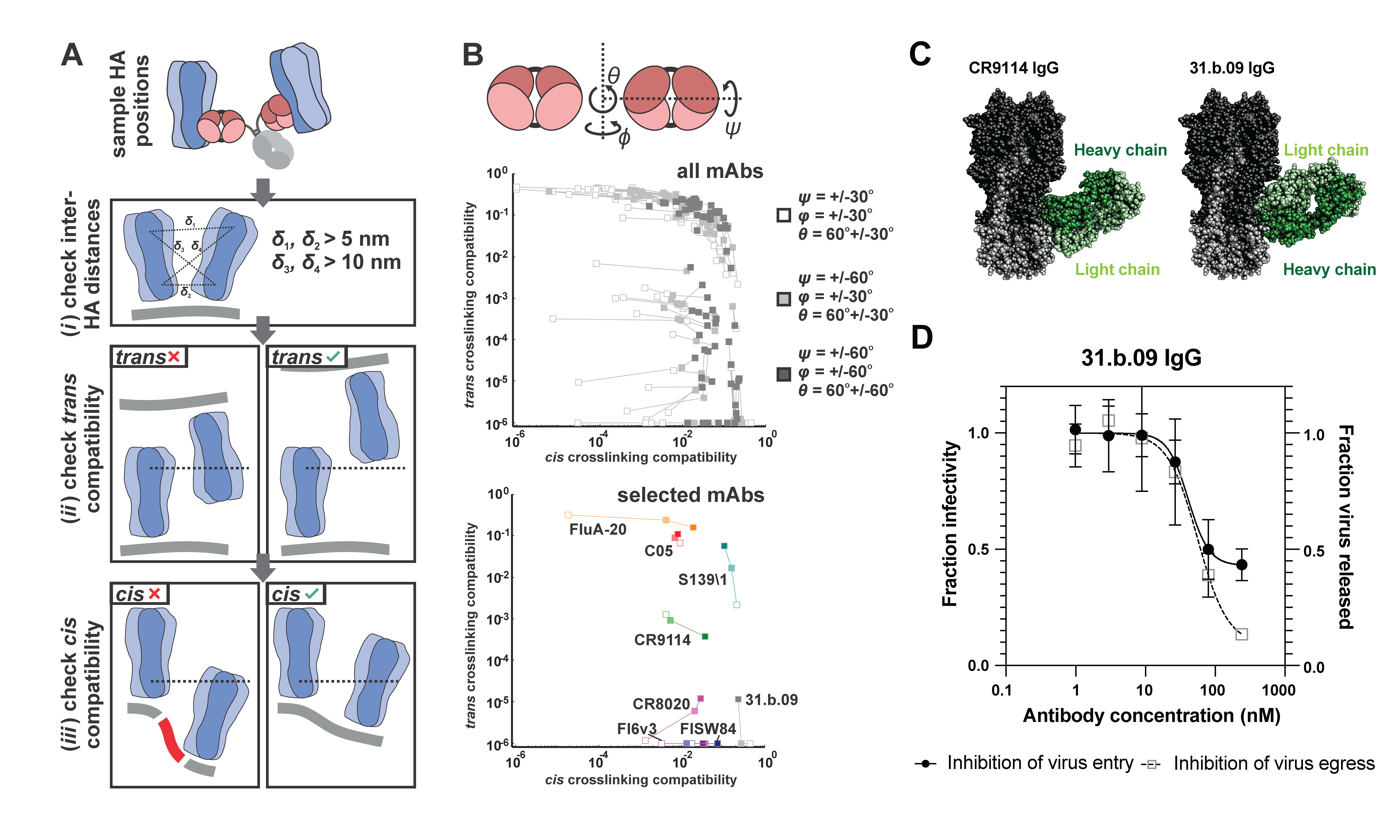

Supplement: Fig. S3 — Predicting cis and trans crosslinking with a structure-based model. [file jvi.01398-23-s0003.tif]
