# Supplementary material for: Antibody inhibition of influenza A virus assembly and release
Source: J Virol. 2024 Jan 5;98(2):e01398-23. doi: 10.1128/jvi.01398-23 (PMC10878280; doi:10.1128/jvi.01398-23)
Supplement: Supplemental legends and Table S1 — Legends for supplemental figures. Table S1. Inhibition of viral entry and release for S139\1 and C05 against different viral strains. [file jvi.01398-23-s0004.docx]

**Figure S1. Virus morphology does not strongly affect inhibition of viral egress, but neutralizing antibodies can alter the apparent size of released virions.** (A) Radar plot showing the IC50 values for viral egress for spherical (WSN33 WT) and filamentous (WSN33 M1Ud) IAV strains with the same HA. (B) Radar plot showing IC50 values for viral entry (determined from microneutralization assays) for the same viral strains as in *A*. IC50 values for viral egress and viral entry are obtained from 3 biological replicates. (C) Distribution of fluorescence intensities for individual viral particles shed in the presence or absence of CR8020 IgG. Fluorescence is measured using labeled C05 Fab. (D) Prevalence of viral filaments above different length thresholds released in the presence or absence of CR8020 IgG. Data is combined from 3 biological replicates. *P* values are determined by independent t-tests.

**Figure S2. Binding and neutralization of antibodies with limited epitope access.** (A) Representative images of cells infected by A/WSN/1933 and treated with FluA-20 (top) or FISW84 (bottom) at 60nM for 6 hpi. Cell nuclei are stained for cell visualization. (B) Inhibition of viral shedding by FISW84 IgG for cells infected by IAV with HA from CA09 or WSN33. (C) Inhibition of viral shedding by FluA-20 IgG for cells infected by IAV with HA from CA09 or WSN33. Data in (B) and (C) is normalized to the antibody-free condition. *P values* are determined by independent t-test.

**Figure S3. Predicting *cis* and *trans* crosslinking with a structure-based model.** (A) Schematic illustrating criteria for *cis* or *trans* crosslinking based on sampled positions and orientations of HAs. Candidate positions and orientations are sampled as shown in Figure 6A and described in Methods. (B) Predicted changes in *cis* and *trans* crosslinking propensities for antibodies modeled with different implicit flexibilities. Top: results for three models of flexibility across all analyzed mAbs. Bottom: results for selected antibodies tested in this work. (C) Structure of CR9114 Fab (PDB ID 4FQI) and 31.b.09 Fab (PDB ID 5K9O) bound to HA. (D) Neutralization curves showing inhibition of viral entry and egress by 31.b.09 IgG against virus with CA09 HA and NA (PR8 reassortant). Curves are obtained from 3 biological replicates and normalized to the plateau of the fitted line for direct comparison. Error bars show standard deviations and the fit curve is obtained using the least squares method.

|  | HK68  entry  [nM] | HK68 release [nM] | IC50 ratio (HK68) | WSN33 entry  [nM] | WSN33 release [nM] | IC50 ratio (WSN33) |
| --- | --- | --- | --- | --- | --- | --- |
| S139\1 IgG | 0.066 | 0.868 | **0.076** | 1.221 | 8.961 | **0.136** |
| C05 IgG | 1.050 | 0.870 | **1.207** | 0.245 | 0.199 | **1.231** |

**Table S1. Inhibition of viral entry and release for S139\1 and C05 against different viral strains**. IC50_entry_ and IC50_release_ values against A/WSN/1933 (M1Ud) and A/HK/1968 are determined by least squares fit from data in Figure 5B. Both WSN33 M1Ud (labelled here as WSN33) and HK68 viral strains exhibit filamentous phenotype to facilitate comparison.

**Table S2. Predicted cis and trans crosslinking propensities from HA-Fab structures**. Data is plotted in Figure 6C and in Figure S3B. Scores less than 10^-6^ are set to 10^-6^ for visualization on a log scale. Please see *supplemental_table_2.xlsx* for complete data.
